# Melatonin Alleviates Cardiac Function in Sepsis-Caused Myocarditis *via* Maintenance of Mitochondrial Function

**DOI:** 10.3389/fnut.2021.754235

**Published:** 2021-10-11

**Authors:** Liyang Chen, Qing Tian, Zhiguang Shi, Yu Qiu, Qiulun Lu, Chao Liu

**Affiliations:** ^1^Key Laboratory of Cardiovascular and Cerebrovascular Medicine, Collaborative Innovation Center for Cardiovascular Disease Translational Medicine, Nanjing Medical University, Nanjing, China; ^2^Intensive Care Unit of Wuhan Asia Heart Hospital, Wuhan, China; ^3^Center for Molecular and Translational Medicine, Georgia State University, Atlanta, GA, United States; ^4^Hubei Key Laboratory of Diabetes and Angiopathy, Hubei University of Science and Technology, Xianning, China

**Keywords:** melatonin, sepsis-induced myocarditis, mitochondria, ROS, cardiac function

## Abstract

Melatonin (N-acetyl-5-methoxytryptamine) has been shown to have a cardioprotective effect against myocarditis. However, the mechanisms underlying the protective role of melatonin (MLT) in sepsis-induced myocarditis are yet to be revealed. In this study, MLT was administrated to mice, 14 days before cecal ligation puncture surgery. Echocardiography results showed that MLT alleviated cardiac dysfunction in sepsis-induced myocarditis. Furthermore, MLT reduced cardiac inflammation by inhibiting the expression of *Il-1*α, *Il-1*β, *Il-6*, and *Mcp-1* messenger RNA (mRNA) levels. The RNA sequencing (RNA-seq) assays with heart tissues showed that MLT maintains the mitochondrial function in sepsis-caused myocarditis. Additionally, the production of reactive oxygen species (ROS) in heart tissues was suppressed by MLT. Taken together, in evaluating the therapeutic effect of MLT on sepsis-induced myocarditis, the results showed that MLT alleviated cardiac damage by regulating mitochondrial function and mitochondrial ROS.

## Introduction

Sepsis represents a syndrome of physiologic, pathologic, and biochemical abnormalities caused by a systemic response to infection, which can result in dysfunctions of the lung, brain, liver, kidney, and heart (1, 2). Myocarditis is a common complication that accounts for deaths in patients with sepsis, and its incidence is increasing yearly. Sepsis-induced myocarditis is present in half of all the patients with septic shock (3) and is characterized by the significant impairment of the left ventricular (LV) systolic and diastolic function (4). Although there have been major advances in the understanding the pathophysiology of sepsis over the past 20 years, much of the associated mortality and morbidity are results of severe derangements in the cardiovascular system (5).

Myocarditis is an independent prognostic risk factor for septic patients. Although the aim therapies for target individual pathways are attractive in theory, these solutions have yet to be proven effective in the treatment of sepsis (6). Because of the shortage of therapeutic strategies for sepsis-induced myocarditis, effective prevention is especially critical. Previous studies revealed that the activation of the neuregulin-1/ErbB signaling axis is beneficial for protection from sepsis-induced myocarditis (7). Mitochondrial dysfunction is also involved in the pathogenesis of sepsis, and the subsequent generation and accumulation of reactive oxygen species (ROS) are associated with the development of mitochondrial dysfunction. The cardiac function can be improved by inhibiting the process of oxidative stress (8). However, there are still no effective pharmacological strategies to treat or even reverse sepsis-induced myocarditis.

There are many factors involved in the pathological process of sepsis-induced myocarditis, such as mitochondrial dysfunction, oxidative stress, pathogen-associated molecular patterns (PAMPs), damage-associated molecular patterns (DAMPs), nitric oxide, and others (6, 9–11). Under pathological conditions with sepsis or other stresses, mitochondrial homeostasis is always imbalanced. In this process, ROS are generated as by-products of the incomplete four-electron reduction of molecular oxygen to water. Reactive oxygen species can be activated avidly by the surrounding molecules in an indiscriminate fashion with both highly reactive and short-lived ways that cause damage if not cleared (12). Once mitochondrial dysfunction persists, ROS are generated in the cardiomyocytes from septic hearts, while oxidative stress induced by ROS mediates mitochondrial damage, which accelerates mitochondrial dysfunction (13). The production of ROS in the mitochondria is essential for normal cardiac function and survival, and there is a need for a new drug that supplements it.

Melatonin, namely, N-acetyl-5-methoxytryptamine, is synthesized centrally in the pineal gland of vertebrates (especially mammals) and can be locally synthesized in several types of cells and tissues (14). Melatonin (MLT) is considered an antioxidant and an anti-inflammatory factor, which also optimizes mitochondrial function. Its antioxidant efficacy is great compared with vitamins C and E (15). High MLT concentrations can be found in the mitochondria in many cell types, where it is proposed to induce sirtuins and inhibit mitochondrial ROS production (16). The existence of MLT could even help improve the success rates of organ transplants by reducing the production of ROS (17). The mechanism by which MLT inhibits oxidative stress in the heart remains unclear.

In this study, we demonstrated that MLT attenuated heart dysfunction in sepsis-induced injury in heart tissues. The results showed that MLT suppressed the sepsis-induced increase an oxidative response, resulting in the declined levels of inflammatory factors. Our work revealed that MLT could be a promising strategy for myocarditis in sepsis.

## Methods and Materials

### Animals

C57BL/6 mice (male, aged 8 weeks) were obtained from the Animal Core Facility of Nanjing Medical University, China. They were housed individually in cages under hygienic conditions with a constant room temperature (23 ± 1°C) and humidity (30–40%). They were also maintained on a 12 h light/dark cycle room, with standard mouse chow and water *ad libitum*. All animal experiments were approved by the Animal Care and Use Committee of Nanjing Medical University and were conducted in accordance with the National Institutes of Health Guide for the Care and Use of Laboratory Animals (IACUC-2005028).

### Cecal Ligation Puncture

The mice subjected to cecal ligation puncture (CLP) treatment were grouped as follows: sham group (*n* = 6), sham + MLT group (*n* = 6), CLP group (*n* = 6), and CLP + MLT group (*n* = 6). For the drinking water, MLT (CAS No. 73-31-4, MCE) was dissolved in 0.1% ethanol, and the final MLT concentration was at 25 μg/ml. The sham group animals were given the drinking water with 0.1% ethanol. MLT was administered for a total of 14 days, and on day 15, the mice were operated on with CLP. The CLP was performed as described previously (18). Briefly, the mice were deeply anesthetized with inhaled isoflurane using an anesthetic vaporizer. The cecum was exposed by performing a 1- to 2 cm midline incision on the anterior abdomen; the distal half of the cecum was ligated and punctured once with a 19 G needle in the ligated segment. The cecum was then placed back into the abdomen, 1 ml of sterile saline (pyrogen-free 0.9% NaCl) was administered subcutaneously, and the incision was closed with a suture needle. The hearts were collected 24 h after the treatment.

### Dihydroethidium Staining

To assess the oxidant levels in the samples, the sections of the frozen heart tissues from mice were stained with DHE (S0063, Beyotime, Shanghai, China) for 30 min. A fluorescence microscope was used to observe and obtain images. The fluorescence intensity of each group was calculated with ImageJ (version 1.52i, National Institutes of Health, Bethesda, MD, USA) to analyze the ROS levels.

### Immunofluorescence

The immunofluorescence staining of cardiac tissues was accomplished using cardiac paraffin sections. The paraffin sections were dewaxed, rehydrated, and subjected to antigen repair in 10 mM of a citrate buffer (pH 6). After closure with 3% bovine serum albumin (BSA) (Sigma, Burlington, MA, United States), the sections were incubated with a mouse anti-CD68 (1:100, MCA1957, Bio-Rad, California, United States) in a humidified chamber at 4°C overnight, followed by incubation with an Alexa Fluor^®^ 594 secondary antibody (1:500, Invitrogen, Waltham, Massachusetts, United States) for 2 h at room temperature, followed by DAPI (ThermoFisher, Waltham, Massachusetts, United States) to stain the nuclei, and images were acquired with a fluorescence microscope (BX53, Olympus, Tokyo, Japan).

### Histology Staining

The heart samples from the mice were fixed in 10% phosphate-buffered formalin for 48 h at 4°C, embedded in paraffin. The samples were sliced into 4-μm-thick sections. These sections were stained with hematoxylin-eosin (G1005, Servicebio, Wuhan, China). Briefly, the heart sections were stained with hematoxylin for 10 min and washed with running water for 5 min. Next, the sections were placed into 0.5 % acid alcohol for 10 s for differentiation and then washed with running water for 5 min. The sections were then stained with eosin for 20 s. After washing for 5 min, all the stained sections were dehydrated in a graded series of 70, 80, 90, and 100% ethanol and then cleared in xylol for 15 min. The slices were then observed under a light microscope.

### RNA Isolation and Real-Time Quantitative PCR

For the tissues, the total RNA was extracted as previously described, using a Trizol reagent (R401-01, Vazyme, Nanjing, China), then reverse-transcribed into complementary DNA (cDNA) using an RT SuperMix (R323-01, Vazyme), following the protocol of the manufacturer. Quantitative real-time PCR (qPCR) assays were performed with QuantStudio 5 (Appliedbiosystems, Thermo Fisher Scientific), using an SYBR Green Mix (Q131-02, Vazyme, China). Gene (*Il-1*α, *Il-1*β, *Il-6, Mcp-1, Nox2*, and *Sod2*) expression was normalized to *18S* or *GAPDH* messenger RNA (mRNA). The levels of the target genes were analyzed using the 2^−Δ*Δct*^ method. The primers used are listed in Supplementary Table 1.

### Echocardiography

A Vevo 2100 High-Resolution Micro-Ultrasound System (FUJIFILM Visual Sonics Inc., Toronto, Canada) was used to determine heart function and ventricular dimensions. The mice were anesthetized with 1.5% isoflurane and placed on a heating table in the supine position. Two-dimensional (2D) and M-mode images were recorded in a short-axis view from the mid-left ventricle at the tips of the papillary muscles. The left ventricular end-systolic diameter (LVIDs) was measured. The left ventricular ejection fraction (LVEF) and left ventricular fractional shortening (LVFS) were calculated from the LV dimensions in the 2D short-axis view. All echocardiography procedures, including data acquisition and analysis, were performed by a researcher who was blind to the experimental treatments to avoid biases.

### RNA Sequencing and Gene Ontology Analysis

The samples were collected and flash-frozen in liquid nitrogen and, then treated with Trizol at −80°C until RNA extraction. The total RNA from the tissues was extracted using Trizol. To monitor the RNA degradation and contamination, 1.5% agarose gels were used. The RNA purity was checked using a NanoPhotometer^®^ spectrophotometer (Implen, CA, United States). Afterward, the RNA concentration was measured using a Qubit^®^ RNA Assay Kit in Qubit^®^ 3.0 Fluorometer (Life Technologies, Westlake Village, CA, United States). Next, RNA integrity was assessed using the RNA Nano 6000 Assay Kit of the Agilent Bioanalyzer 2100 system (Agilent Technologies, Santa Clara, CA, United States). These processes were operated by Wuhan Frasergen Genomic Medicine Co., Ltd. The raw reads were filtered using trim-galore, and the clean reads were aligned to mm10 by Hisat2 (v2.1.0) (19). The genes with a fold change of ≥1.5 and *p*-value of ≤ 0.01 were considered differentially expressed using the edgeR package (20). A Gene Ontology (GO) analysis was conducted to identify overrepresented biological processes (21, 22).

### Mitochondrial Function and Antioxidant Status

Approximately 100–150 mg fresh heart tissues were weighed, rinsed with cold normal saline to clean the blood, blotted with filter paper, and then cut into pieces. The extraction of mitochondria in the heart was performed using assay kits, according to the instructions of the manufacturer (SM0020, Solarbio, Beijing, China). The concentrations of mitochondrial isocitrate dehydrogenase (ICDHm, BC2165, Solarbio), succinate dehydrogenase (SDH, BC0955, Solarbio), NADP-malate dehydrogenase (NADP-MDH, BC1055, Solarbio), NADH oxidase (NOX, BC0635, Solarbio), and NADPH-cytochrome C reductase (NCR, BC2725, Solarbio) were measured according to the commercial detection kits. The activities of superoxide dismutase (SOD, BC0175, Solarbio), catalase (CAT, BC0205, Solarbio), glutathione (GSH, BC1175, Solarbio), glutathione s-transferase (GST, BC0355, Solarbio), and glutathione peroxidase (GPx, BC1195, Solarbio) were performed using assay kits, according to the instructions of the manufacturer.

### Statistics

Statistical analyses were performed using the Prism 8 (GraphPad) software. All data were expressed as means ± SD. Two-group comparisons were analyzed using Student's *t*-test or a non-parametric Wilcoxon rank test whenever appropriate. *p* < 0.05 was considered significant.

## Results

### Melatonin Alleviates Cardiac Dysfunction in Sepsis-Induced Myocarditis

**Figure 1:**
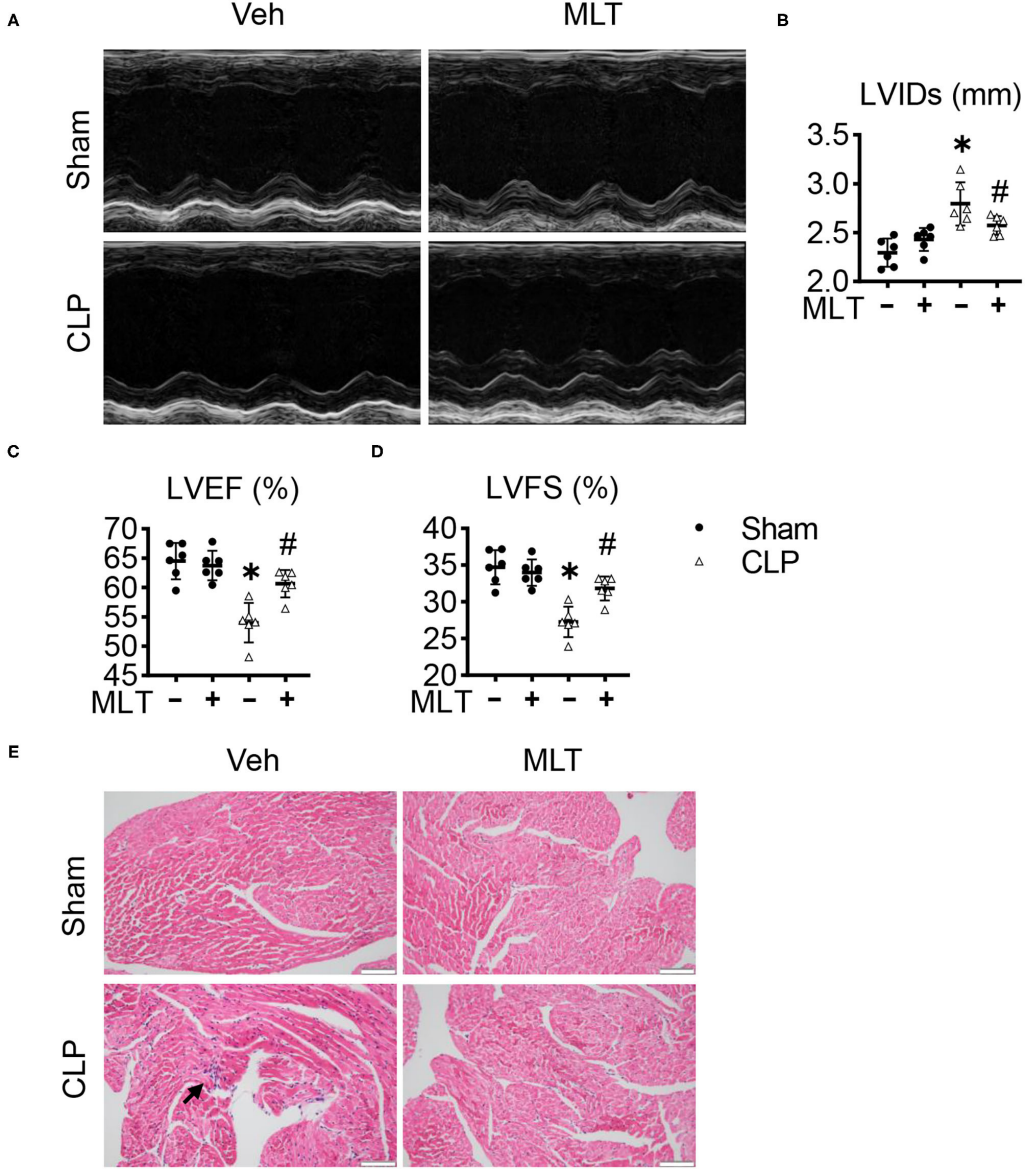
Melatonin alleviates cardiac dysfunction in sepsis-induced myocarditis. Eight-week old mice were treated with melatonin (MLT) (25 μg/ml) dissolved in drinking water for 14 days. **(A)** Representative echocardiogram of the cardiac function of mice (M-mode) for each condition. **(B–D)** Quantification of the left ventricular end-systolic diameter (LVIDs, mm), left ventricular ejection fraction (LVEF, %), and left ventricular fractional shortening (LVFS, %). **(E)** Representative images of the HE staining of the heart tissues (scale bar = 100 μm), the black arrow indicates the inflammatory infiltration area. Values are presented as means ± SD, *vs. sham, *p* < 0.05; ^#^vs. CLP, *p* < 0.05.

We detected the therapeutic effects of MLT for cardiac function during the process of myocarditis in the CLP mouse model, a typical and widely accepted mouse model for sepsis. Echocardiography showed that it was consistent with previous reports that LVIDs were augmented in CLP-induced mice (Figures 1A,B). Compared with the sham group, both LVEF and LVFS in the mice with CLP surgery were decreased (Figures 1C,D). Conversely, MLT significantly improved cardiac function marked by the recovery of LVEF, LVFS, and LVIDs (Figures 1A–D). Hematoxylin and eosin staining the sections from the heart tissues showed that the CLP group exhibited a significant level of inflammatory cell infiltration and MLT protected the heart from inflammation damage (Figure 1E). The results of CD68 immunofluorescence staining also revealed that MLT could reduce immune cell infiltration (Supplementary Figure 1). These results indicate that MLT could maintain cardiac function in sepsis-induced myocarditis.

### Melatonin Relieves Cardiac Inflammation in Sepsis-Induced Mice

**Figure 2:**
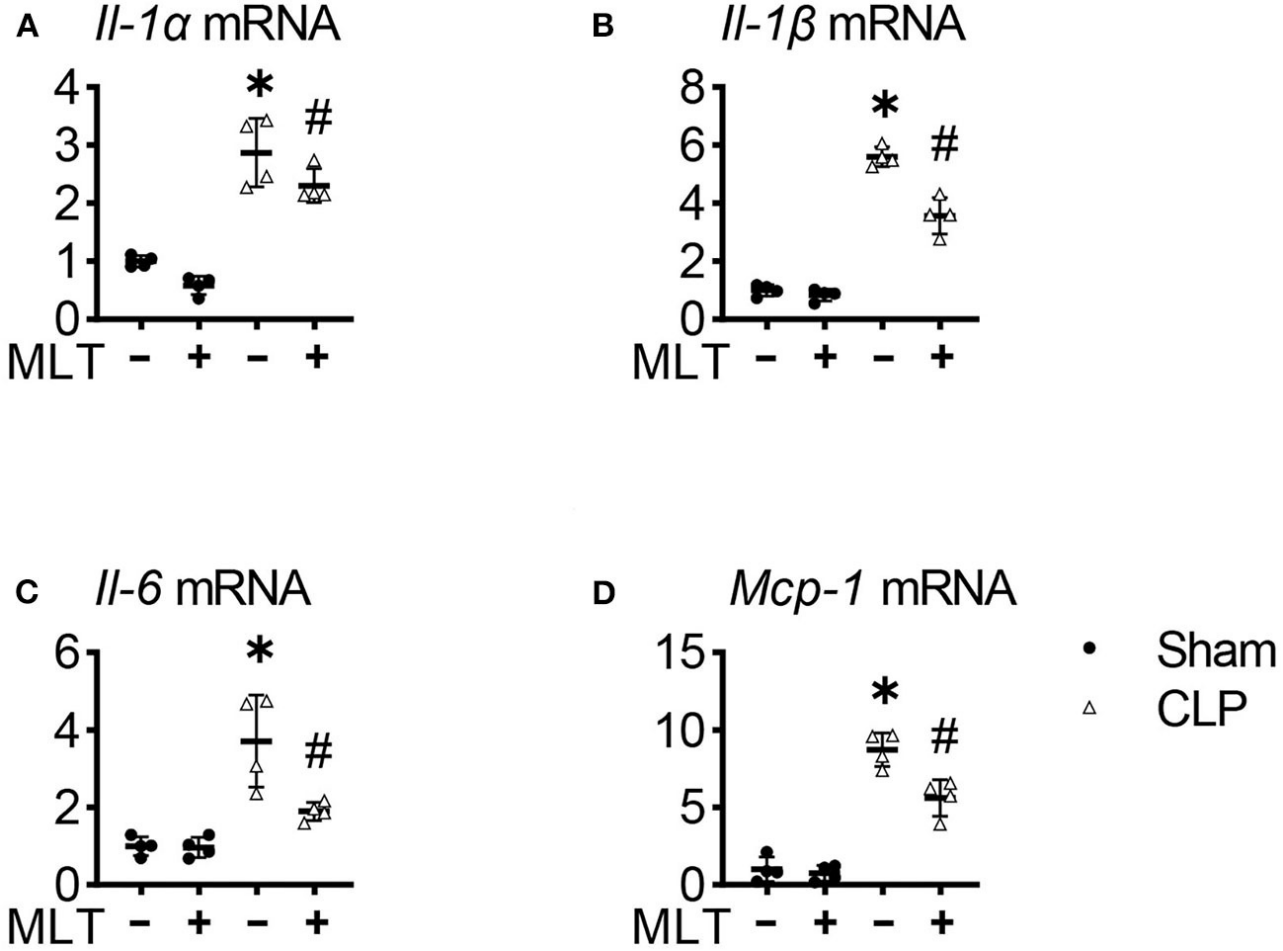
Melatonin relieves cardiac inflammation in sepsis-induced mice. The heart tissue inflammatory cytokines *Il-1*α **(A)**, *Il-1*β **(B)**, *Il-6* **(C)**, and *Mcp-1* **(D)** mRNA levels were measured using real-time quantitative PCR (RT-qPCR). Values are presented as means ± SD, *vs. sham, *p* < 0.05; ^#^vs. CLP, *p* < 0.05.

Considering the inflammation involved in the process of sepsis-induced myocarditis, we detected levels of inflammatory response in sepsis mice after MLT treatment. Real-time PCR assays were performed with the heart tissues from the mice, showing that the mRNA expression levels for pro-inflammatory factors, including *Il-1*α, *Il-1*β, *Il-6*, and *Mcp-1*, were increased in the CLP group compared with the sham group (Figures 2A–D). The elevated levels of these pro-inflammatory factors were significantly suppressed after MLT treatment. Taken together, these results show the therapeutic role of MLT in anti-inflammatory response as verified in the CLP-caused myocarditis.

### Melatonin Ameliorates Mitochondrial Dysfunction in Sepsis-Induced Myocarditis

**Figure 3:**
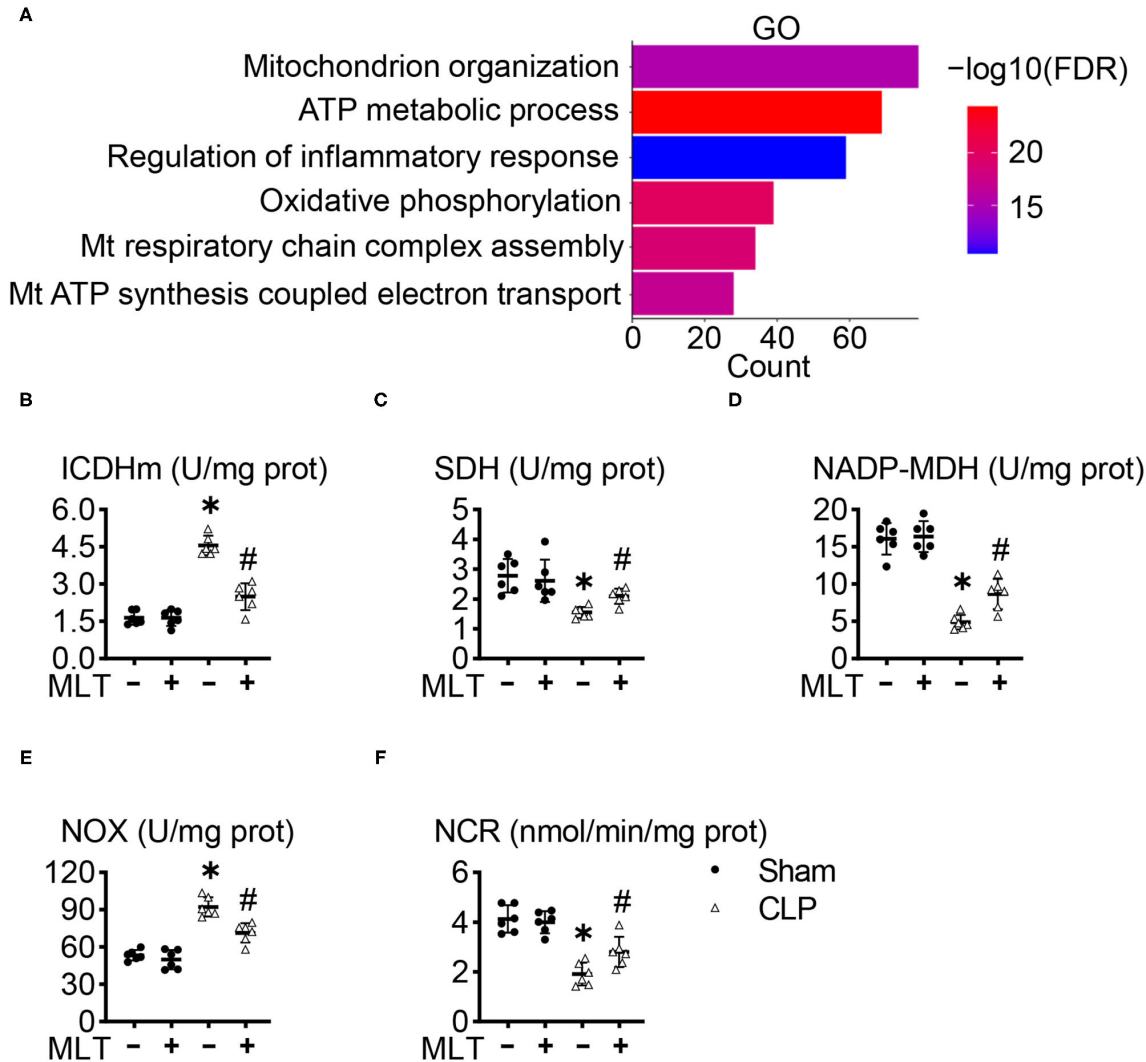
Melatonin ameliorates mitochondrial dysfunction in sepsis-induced myocarditis. **(A)** Gene ontology (GO) enrichment for the regulated genes between the cecal ligation puncture (CLP) group and CLP + MLT group. The effects of MLT on the cardiac mitochondrial levels of mitochondrial isocitrate dehydrogenase (ICDHm) **(B)**, succinate dehydrogenase (SDH) **(C)**, malate dehydrogenase (MDH) **(D)**, NADH oxidase (NOX) **(E)**, and NADPH-cytochrome C reductase (NCR) **(F)**. Values are presented as means ± SD, *vs. sham, *p* < 0.05; ^#^vs. CLP, *p* < 0.05.

To explore how MLT alleviates cardiac dysfunction in sepsis-induced myocarditis, we performed an RNA sequencing (RNA-seq) assay with the heart tissues, and the GO analysis showed that MLT affects the ATP metabolism process, one typical pathway reflecting the function of mitochondria (Figure 3A; Supplementary Figure 2). Afterward, we determined the expression levels and activities for some enzymes in mitochondria. The concentration of ICDHm) was augmented, while those for SDH and NADP-MDH diminished in the CLP group, but the condition of the MLT-treated mice with CLP improved (Figures 3B–D). These results indicate that MLT ameliorates the impaired tricarboxylic acid (TCA) cycle in the mitochondria. Although the tricarboxylic acid cycle (TAC) cycle was impaired in response to sepsis, MLT could transfer the acetyl CoA into TAC for glycolysis. MLT also reduced the expression of NOX while the expression of NCR was elevated (Figures 3E,F). As vital members of the electron transport chain, the variation of NOX and NCR revealed the protective function of MLT in maintaining the normal operation of the mitochondrial respiratory chain. Taken together, MLT could protect mitochondrial function in sepsis-induced myocarditis.

### Melatonin Modulates Mitochondrial Antioxidant Status in the Heart After CLP

**Figure 4:**
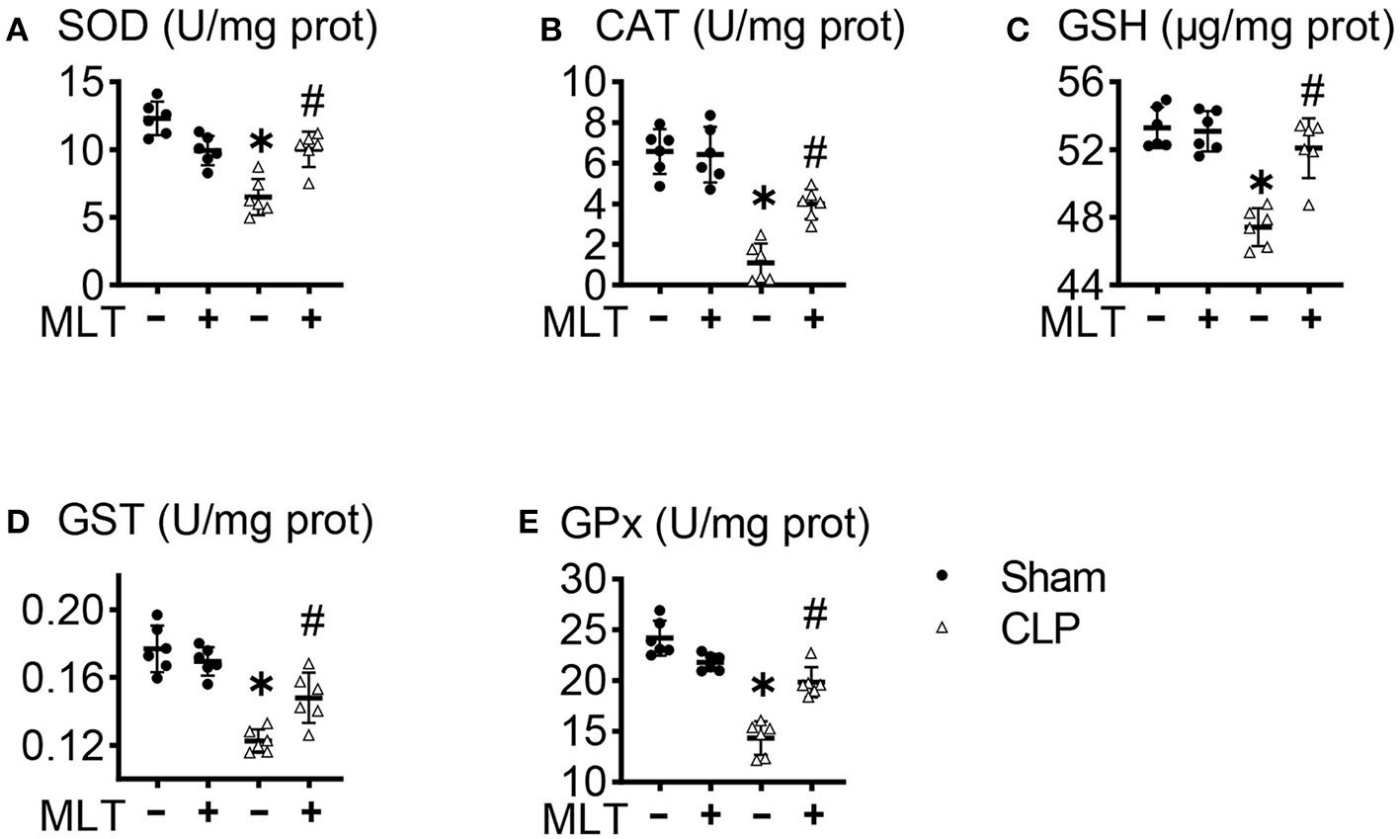
Melatonin modulates mitochondrial antioxidant status in the heart after CLP. Effects of MLT on the cardiac mitochondrial antioxidant levels of superoxide dismutase (SOD) **(A)**, catalase (CAT) **(B)**, glutathione (GSH) **(C)**, glutathione s-transferase (GST) **(D)**, and glutathione peroxidase (GPx) **(E)**. Values are presented as means ± SD, *vs. sham, *p* < 0.05; ^#^vs. CLP, *p* < 0.05.

One effector production for mitochondrial dysfunction is ROS accumulation. In the heart tissues from the CLP group, the activities of SOD were decreased, and MLT impaired this (Figure 4A). The same results for CAT levels were observed (Figure 4B). Additionally, the amounts of GSH, GST, and GPx declined in the CLP group compared with the sham group. Consistently, MLT restored the impaired ability of antioxidant response (Figures 4C–E).

### Melatonin Reduces ROS in CLP-Induced Mice

**Figure 5:**
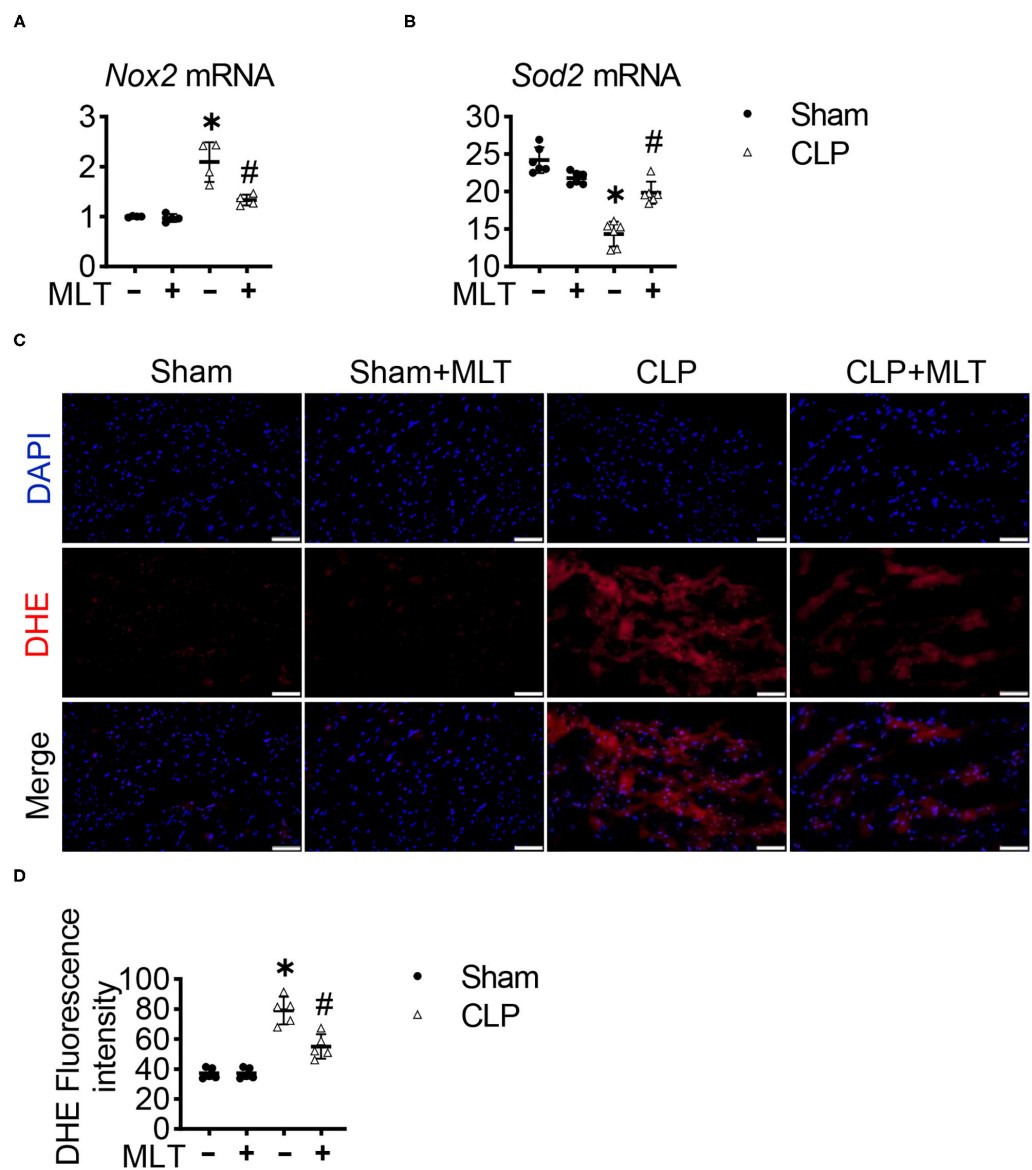
Melatonin reduces reactive oxygen species (ROS) in CLP-induced mice. *Nox2* **(A)** and *Sod2* **(B)** mRNA levels are measured using RT-PCR. **(C)** Representative images of dihydroethidium (DHE) staining of the heart tissues (scale bar = 50 μm). **(D)** The ROS production was quantified and presented as the mean fluorescence intensity. Values are presented as means ± SD, *vs. sham, *p* < 0.05; ^#^vs. CLP, *p* < 0.05.

To further confirm whether MLT could abolish the accumulation of ROS production in CLP-induced heart failure, we also detected the production of ROS in heart tissues. To do this, RT-PCR assays were performed, showing that MLT repressed the CLP-induced increase in the mRNA level of a typical oxidative factor, *Nox2* (Figure 5A), which is the major subtype for the Nox family in the heart. Meanwhile, MLT repressed the reduction of *Sod2* mRNA levels caused by myocarditis (Figure 5B). DHE fluorescent probe was used to detect the ROS levels in the cardiac tissues from the different groups of mice. Compared with the CLP group, the level of ROS in the heart was reduced after MLT treatment (Figures 5C,D). These results show that MLT could reduce cardiac injury by abolishing ROS accumulation.

## Discussion

This study found that MLT can play a protective role in myocarditis caused by sepsis. Mechanistically, MLT alleviated the inflammatory response induced by sepsis, restored mitochondrial function, and scavenged ROS productions. Our results indicate that MLT is a potential therapeutic strategy for sepsis-caused myocarditis in clinics.

Myocarditis is a major complication of sepsis and has a negative impact on the survival of patients (23). Evidence reveals that there are several molecular mechanisms underlying sepsis-caused heart failure, such as mitochondrial dysfunction, the accumulation of ROS productions, and so on. Sepsis causes cardiac mitochondrial dysfunction by the elevation of oxidative stress, as shown in the accumulation of ROS productions and the reduction of antioxidant gene expression. Oxidative stress causes mitochondrial dysfunction and further increases the production of ROS (24). The elimination of mitochondrial ROS can be achieved *via* enzymatic and non-enzymatic antioxidants. The typical enzymes for antioxidative response include GPx, CAT, and SOD. Herein, MLT increased the levels of GPx, CAT, and SOD in the heart tissues from the sepsis-affected mice. Some cases revealed that mitochondria-targeted ROS scavengers like mitochondrial Q were cardioprotective (25). The anesthetic agent, propofol, explores its cardioprotective effect by scavenging free radicals (26). Furthermore, NOX inhibitors were also indicated to attenuate disease-related ROS formation selectively to protect cardiac function (27). In this study, MLT can also manifest an antioxidative effect by alleviating the mitochondrial ROS-caused damage to protect cardiac function. A myocarditis mouse model was established by CLP surgery, and cardiac dysfunction was ameliorated by MLT. The results revealed that CLP causes the worst injury, whereas MLT alleviated the CLP-caused injury and cardiac dysfunction *via* its anti-inflammatory and antioxidative role.

Our studies revealed that the anti-inflammatory function of MLT was *via* abolishing the accumulation of ROS productions. Melatonin is a unique molecule with a variety of molecular functions. It targets MLT receptors located in the plasma membrane or mitochondria or is independent of the receptors. By acting as an antioxidant or free radical scavenger, it can stimulate and inhibit osteoclasts and improve bone density (28). In *Streptococcus pneumonia*-infected rabbits, MLT significantly increases the activity of SOD and reduces the nitrite concentrations to resist oxidative stress (29). Additionally, MLT can enhance intracellular GSH levels by stimulating γ-glutamylcysteine synthase to protect the nervous system from oxidative damage (30). These studies indicate that MLT is a highly effective free radical scavenger and a powerful effect of antioxidant. Furthermore, MLT acts as an antioxidant by elevated antioxidative enzyme expression. Our studies also revealed that the cardioprotective function of MLT was realized by abolishing the accumulation of ROS productions.

As is commonly known, the role of MLT is mediated with the corresponding receptor of MLT. MLT is an indoleamine with lipophilic characteristics that goes across cell membranes and easily gets access to different subcellular compartments. For example, MLT happens to interact with lipid bilayers around the mitochondria, stabilizing the mitochondrial inner membranes and enhancing electron transport chain activity. It has been reported that MLT regulates the activities of complexes I and IV by interacting with the components of the electron transport chain to increase electron flow, and consequently, ATP production (31). As is known, ROS are synthesized as a subproduct of the mitochondrial electron transport chain. There is a large body of evidence showing that MLT is a major scavenger of oxygen-based reactive molecules. Furthermore, MLT provokes this effect at both physiological and pharmacological concentrations. Several of its metabolites can also detoxify free radicals and derivatives (32).

In conclusion, the MLT-mediated antioxidative function acts against sepsis-induced myocarditis by affecting the mitochondrial dysfunction induced by mitochondrial ROS. The data of this study show that MLT administration mitigated the activation of inflammation in sepsis by suppressing the production of mitochondrial ROS. An in-depth understanding of this mechanism is needed to provide direction for the treatment of sepsis-induced myocarditis in the future.

## Data Availability Statement

The datasets presented in this study can be found in online repositories. The names of the repository/repositories and accession number(s) can be found below: https://www.ncbi.nlm.nih.gov/geo/, GSE178780.

## Ethics Statement

The animal study was reviewed and approved by Animal Care and Use Committee of Nanjing Medical University.

## Author Contributions

QL and LC designed the study. LC and QT conducted the searches. ZS and YQ analyzed the data. QL, CL, and LC wrote the manuscript. All authors contributed to the article and approved the submitted version.

## Funding

This project was supported by grants from the National Natural Science Foundation of China (81970414 to QL and 81870173 to CL) and the Natural Science Foundation of the Jiangsu Higher Education Institutions of China (19KJA350001 to QL).

## Conflict of Interest

The authors declare that the research was conducted in the absence of any commercial or financial relationships that could be construed as a potential conflict of interest.

## Publisher's Note

All claims expressed in this article are solely those of the authors and do not necessarily represent those of their affiliated organizations, or those of the publisher, the editors and the reviewers. Any product that may be evaluated in this article, or claim that may be made by its manufacturer, is not guaranteed or endorsed by the publisher.
