# Supplementary material for: Melatonin Alleviates Cardiac Function in Sepsis-Caused Myocarditis via Maintenance of Mitochondrial Function
Source: Front Nutr. 2021 Oct 11;8:754235. doi: 10.3389/fnut.2021.754235 (PMC8542660; doi:10.3389/fnut.2021.754235)
Supplement: Supplementary file 1 [file Data_Sheet_1.PDF]

Supplementary Materials

Melatonin alleviates cardiac function in sepsis-caused myocarditis via maintenance of mitochondrial function

Liyang Chen[1,#], Qing Tian[2,#], Zhiguang Shi[1], Yu Qiu[3], Qiulun Lu[1,*], Chao Liu[4,*]

1. Key Laboratory of Cardiovascular and Cerebrovascular Medicine, Collaborative Innovation Center for Cardiovascular Disease Translational Medicine, Nanjing Medical University, Nanjing 211166, China

2. Intensive Care Unit of Wuhan Asia Heart Hospital, Wuhan 430000, China

3. Center for Molecular and Translational Medicine, Georgia State University, Atlanta, GA 30303, USA

4. Hubei Key Laboratory of Diabetes and Angiopathy, Hubei University of Science and Technology, Xianning 437100, China

# These authors contributed equally to this work.

* Correspondence should be addressed to C. L., Hubei Key Laboratory of Diabetes and Angiopathy, Hubei University of Science and Technology, Xianning 437100, China, Phone: (86)- 13971800606, Email: liuchao@hbust.edu.cn; Q. L., Key Laboratory of Cardiovascular and Cerebrovascular Medicine, Nanjing Medical University, Nanjing 211166, China, Phone: (86)-17366385459, Email: qiulunlu@njmu.edu.cn

Supplementary Table 1. Primers for quantitative reverse transcription PCR detection

Name	Forward primer (5'-3')	Reverse primer (5'-3')
Il-1α	CGAAGACTACAGTTCTGCCATT	GACGTTTCAGAGGTTCTCAGAG
Il-1β	GAAATGCCACCTTTTGACAGTG	TGGATGCTCTCATCAGGACAG
Il-6	TCTATACCACTTCACAAGTCGGA	GAATTGCCATTGCACAACTCTTT
Mcp-1	GCATCCACGTGTTGGCTCA	CTCCAGCCTACTCATTGGGATCA
Nox2	ACTCCTTGGGTCAGCACTGG	GTTCCTGTCCAGTTGTCTTCG
Sod2	CCGTCCTTTCCAGCAGTC	GACCTGCCTTACGACTATG
18S	GTAACCCGTTGAACCCCATT	CCATCCAATCGGTAGTAGCG
GAPDH	AGGTCGGTGTGAACGGATTTG	TGTAGACCATGTAGTTGAGGTCA

Supplementary Figure 1

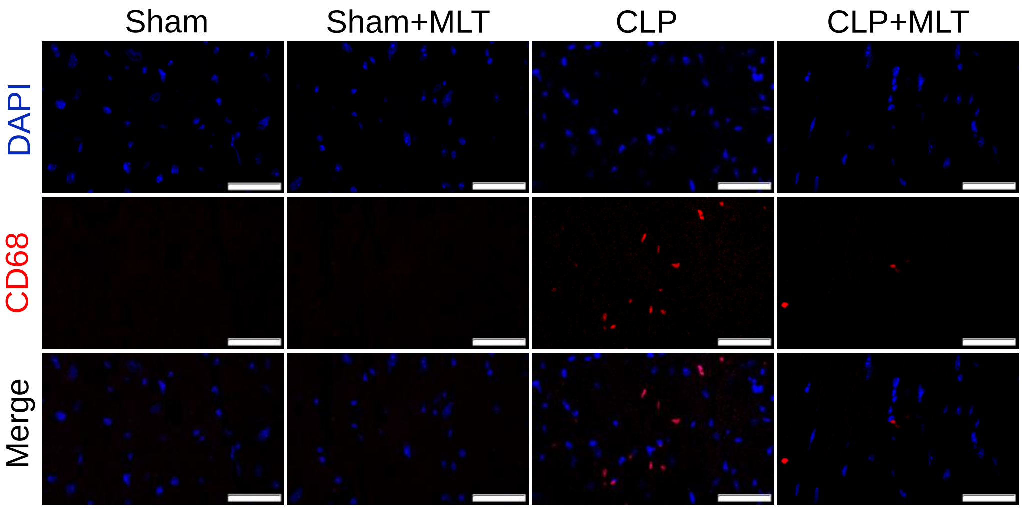

Supplementary Figure 1: Representative images of CD68 immunofluorescence staining of heart tissues (scale bar = 20 μm).

Supplementary Figure 2

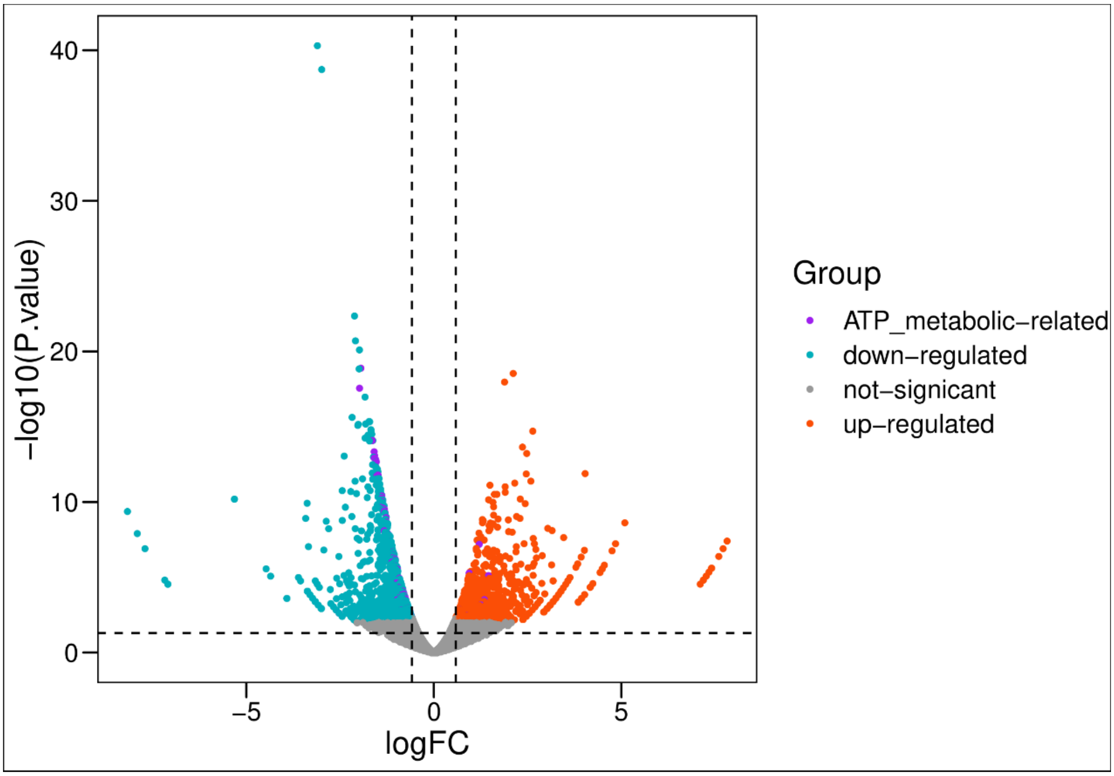

Supplementary Figure 2. Volcano plot for the comparison between the CLP and CLP+MLT groups. The cutoff values fold change >1.5 and FDR < 0.01 were utilized to identify differentially expressed genes. Non-changed genes were shown in grey color. Red color is indicative of up-regulated genes, green is indicative of down-regulated genes and purple is indicative of genes related to ATP metabolism.
